# Midwives' Knowledge, Attitudes, and Practices of Liberal Birthing Position Management During the Second Stage of Labor: A Cross-Sectional Study

**DOI:** 10.1155/jonm/6139992

**Published:** 2025-02-24

**Authors:** Taizhen Luo, Yang Gong, Hua-an Xia, Zhiyan Jiang, Huating Chen, Lingling Gao

**Affiliations:** ^1^Department of Nursing, Guangdong Provincial Key Laboratory of Major Obstetric Diseases, Guangdong Provincial Clinical Research Center for Obstetrics and Gynecology, The Third Affiliated Hospital, Guangzhou Medical University, Guangzhou, China; ^2^Faculty of Education, University of Macau, Macau, China; ^3^Department of Obstetrics and Gynecology, Obstetrics, Guangdong-Hong Kong-Macao Greater Bay Area Higher Education Joint Laboratory of Maternal-Fetal Medicine, The Third Affiliated Hospital, Guangzhou Medical University, Guangzhou, China; ^4^School of Nursing, Guangzhou Medical University, Guangzhou, China; ^5^School of Nursing, Sun Yat-sen University, Guangzhou, China

**Keywords:** attitudes, birthing position, knowledge, midwifery, second stage of labor

## Abstract

**Objective:** To investigate the knowledge, attitudes, and practices of Chinese midwives regarding liberal birthing position management during the second stage of labor and explore the factors related to their practices.

**Background:** Midwives play a critical role in managing the second stage of labor, during which appropriate delivery posture is essential for promoting normal birth and improving childbirth experiences.

**Methods:** A cross-sectional study was conducted in Guangdong Province, China, from April to May 2023. Three hundred and sixty midwives completed the questionnaire on knowledge, attitude, and practice of liberal birthing position management during the second stage of labor. *SPSS 29.0* was used to analyze the data. Descriptive statistics, independent sample *t*-test, one-way ANOVA, Pearson correlation analysis, and multiple linear regression analysis were performed. *p* < 0.05 was considered statistically significant.

**Results:** The midwives' mean knowledge total score was high, with an 83.1% rate of correct answers. The midwives had a lower moderate level of attitude with a mean total score of 43.06 ± 9.08 out of 76 and a low level of the Worry dimension of attitude (19.86 ± 7.92 out of 48). The midwives had a moderate level of practice with a mean total score of 3.03 ± 1.85 out of 6. The scores of knowledge, attitude, and practice were pairwise positively correlated (*p* < 0.001). The multiple linear regression analysis showed that knowledge, attitude, having participated in training on maternal birthing positions, having experiences in clinical teaching, hospital grade, and hospital category were the predictors of midwives' practice of liberal birthing position management during the second stage of labor (*p* < 0.05).

**Conclusion:** Midwives' attitudes and practices of liberal birthing position management during the second stage of labor need to be improved, and participating in training and undertaking clinical teaching tasks are crucial for them to improve their practices in this area.

**Implications:** Midwifery managers need to pay more attention to the professional education and training of midwives and should establish organizations that are specifically designed to support midwives' continuous learning and professional development. These organizations can offer targeted training programs, create collaborative learning environments, and facilitate the sharing of best practices among midwives. Nursing educators should be familiar with and in line with international standards, emphasizing upgrading midwifery teaching materials and establishing a standardized on-the-job training system.

## 1. Background

Maternal birthing positions directly affect the 4Ps (Passage, Passenger, Power, and Psyche), which, in turn, affect maternal and infant outcomes [1, 2]. The second stage of labor is frequently the most challenging and stressful for both the mother and the fetus, as well as for midwives who are assisting with the delivery [3]; it can be divided into a passive phase, an active phase, and the baby's actual birth when the baby emerges [4]. According to whether the sacrum supports the body weight, birthing positions during the second stage of labor can be classified into nonflexible sacrum and flexible sacrum positions [5]. The flexible sacrum positions include standing, squatting, kneeling, sitting upright, hands-and-knees, and lateral position. The nonflexible sacrum positions include semirecumbent, semisitting, lithotomy, and dorsal positions.

Appropriate birthing position management during the second stage of labor is critical for promoting normal birth and improving childbirth experiences [6, 7]. Evidence suggests that liberal birthing positions in the second stage of labor can help the labor process without medical intervention [8, 9]. Liberal birthing positions refer to women who can ambulate and change their positions freely [10]. A recent meta-analysis shows that the liberal birthing position can significantly shorten the duration of the second stage of labor [2, 10]. Moreover, multiple studies have shown that the liberal birthing position in the second stage of labor is associated with fewer interventions, less pain, and a more satisfying delivery experience than the traditional supine/lithotomy position [6, 9, 11–13]. International guidelines recommend that midwives should support women to move liberally and change positions freely and discourage them from adopting supine or semisupine/lithotomy positions during the second stage of labor [14, 15]. Therefore, liberal birthing position in this study refers to the flexible sacrum positions, the exclusion of fixed single position, and traditional lithotomy position during the second stage of labor.

In mainland China, women usually give birth in secondary or tertiary maternal and child hospitals or general hospitals. Maternal and child hospitals focus on medical and health care services for women and children. General hospitals provide comprehensive medical and health care services. Usually, secondary hospitals have a neonatology department, while tertiary hospitals also have a neonatal intensive care. The practice of liberal birthing positions in labor rooms in mainland China is limited, as well as in many Western countries [6, 16, 17]. A survey of 1213 hospitals in mainland China showed that nearly half of the (47.8%) hospitals do not encourage the adoption of liberal positions during the second stage of labor [18]. Although most pregnant women in mainland China are interested and have a high willingness to assume the liberal birthing position, their preferences are only partially satisfied in practice [19, 20].

The British scientist John Coster first proposed the theory of knowledge, attitude, and practice (KAP) in the 1960s. It indicates that knowledge is the foundation of practice, attitude is the driving force, and knowledge can influence attitude. In this regard, midwives' knowledge and attitude may relate to their practice of liberal birthing position management during the second stage of labor. Furthermore, Musie, Peu, and Bhana-Pema [21] suggested that a lack of necessary skills and professional training were the barriers to using alternative birthing positions. In mainland China, liberal birthing positions are not taught during midwives' undergraduate training and continued education [22].

Midwives have the responsibility to promote maternal and infant outcomes. It is suggested that all midwives should update their knowledge and actively provide information on birthing positions to women according to their wishes and comfort, providing the best birth experience [23]. Thus, there is a priority and practical need for nurse managers and educators to understand midwives' knowledge, attitudes, and practices. However, the literature shows that studies on the state of knowledge, attitudes/beliefs, and practices in labor/birthing/delivery positions have some reports on pregnant women and none on midwives [24, 25]. Therefore, the present study aimed to investigate the midwives' knowledge, attitudes, and practices of liberal birthing position management during the second stage of labor in mainland China, find out the existing problems, and take targeted improvement measures to promote the practice of liberal birthing position and improve the maternal delivery outcomes and experiences.

## 2. Methods

### 2.1. Study Design, Setting, and Participants

A cross-sectional study was implemented from April to May 2023 under the guidance of the Strengthening the Reporting of Observational Studies in Epidemiology Checklist of cross-sectional studies. Midwives were recruited in Guangzhou, a sub-provincial city in southeastern China. Guangzhou is the capital of the Guangdong Province and has a population of approximately 20 million. In 2021, there are 126 midwifery institutions with 101 secondary and tertiary hospitals in Guangzhou [26] and the birth rate was 11.82% [27].

Because women usually give birth in maternal and child or general hospitals at the secondary level or above, we used convenience sampling to select 15 hospitals (10 tertiary/5 secondary hospitals; 9 general/6 maternal and child hospitals). The midwives who worked in these labor rooms were invited to participate in the study. The inclusion criteria were (1) having a technical qualification certificate maternal and child health care; (2) having worked as a midwife in labor rooms for 1 year or more; and (3) currently working in labor rooms. Accordingly, the exclusion criteria were (1) not present during the data collection period due to going out for further professional development or vacation; (2) coming from other hospitals for professional visits or study.

According to the Kendall method, the sample size is 5–10 times of the survey variables, then, considering 10%–15% of invalid questionnaires, and the final sample size was estimated to be 319–667.

### 2.2. Measures

The Questionnaire on midwifery knowledge, attitude, and practice of liberal birthing position management during the second stage of labor has been developed and validated in Chinese midwives by Luo and Gao [28] and was used in the present study. The detailed items of the Questionnaire are seen in the supporting file.

The Questionnaire on midwives' knowledge of liberal birthing position management during the second stage of labor consists of 28 items and is categorized into four dimensions: Concept (12 items), Functions (4 items), Indications (6 items), and Contraindications (6 items) of liberal birthing position management during the second stage of labor. The answers were divided into “correct” (1 point), “incorrect” (0 points), and “uncertain” (0 points). The total scores range from 0 to 28, with higher scores indicating more knowledge. The reported Cronbach's coefficient of this knowledge questionnaire was 0.89.

The Questionnaire on midwives' attitude toward the liberal birthing position management during the second stage of labor has 19 items and includes two dimensions: Interest and Applying Tendency (7 items), and Worry (12 items). Items were rated on a Likert scale (0 = strongly disagree to 4 = strongly agree). Items 1–7 are scored in the order of positive attitude, that is, 0, 1, 2, 3, 4 in the order of negative attitude to positive attitude; items 8–19 are scored in the negative direction, that is, in the order of negative attitude to positive attitude, the scores are 4, 3, 2, 1, 0. The total scores range from 0 to 76, with a higher score indicating a more positive state. The reported Cronbach's coefficient of the attitude questionnaire was 0.88.

The Questionnaire on midwives' practice of liberal birthing position management during the second stage of labor has three items. Firstly, the midwife was asked, “*Have you helped the women to use liberal birthing position during the second stage of labor?*” The answer “No” will get a 0 score, “Yes, with other midwives' help” will get 1 score; “Yes, by myself” will get 2 scores. The midwife was then asked, “*How many positions have you guided the woman during the second stage of labor?*” The answer 1, 2, 3, and 4 or above will get 0, 1, 2, and 3 scores, respectively. The third question was “*Which position do you often help the woman to use in the phase of the actual birth of the baby when the baby actually emerges?*” The answer of lithotomy will get a 0 score, and the other positions including lateral lying, sitting, squatting, hands and knees, and standing get a 1 score. The total score of the practice questionnaire ranges from 0 to 6, with a higher score indicating better midwifery practice.

Sociodemographic data were collected on midwives' age, gender, experience of giving birth, education, major of graduation, job position, experience of clinical teaching, and experience of trained in maternal birthing positions. Information on the sources of knowledge about the liberal birthing position and the type of training desired to learn were also collected.

### 2.3. Ethical Consideration and Data Collection

Ethical approval was obtained from the Institutional Review Boards (IRBs) of The Third Affiliated Hospital of Guangzhou Medical University ([2023] No. 156). This study conformed to the Code of Ethics of the World Medical Association (Declaration of Helsinki). To ensure the accuracy and consistency of the data collection process, a training session was provided to two research assistants (RAs) and then a pilot study was carried out on 20 midwives.

Before data collection, the principal investigator contacted the nurse management staff in the study labor rooms by phone call or WeChat to get permission. Then, the RAs went to the study labor rooms to invite the midwives to participate in the study. The RAs explained the purpose and significance of the investigation and informed the inclusion and exclusion criteria to the midwives on the site. The midwives were also informed of the confidentiality of their data and the right to withdraw from the study. The midwives could freely choose whether to participate in the study. No participation will not affect them. After giving the informed consent, the midwives were asked to complete the questionnaires independently. The completed questionnaires were then returned to the research assistants.

### 2.4. Statistical Analysis

Data were analyzed using SPSS 29.0 for Windows (IBM Corporation, Armonk, NY, USA). Statistical significance was set at 0.05. Descriptive statistics were used to describe midwives' sociodemographic characteristics, knowledge, attitude, and practice of liberal birthing position management during the second stage of labor. Differences in midwifery practice among groups with different sociodemographic characteristics were computed by the independent sample *t*-test or one-way ANOVA. Pearson correlation analysis was used to explore the correlation among knowledge, attitude, and midwifery practice. All the variables with *p* < 0.05 in the above tests were inputted into a multivariate linear regression model to determine the factors independently associated with the midwifery practice of liberal position management in the second stage of labor.

## 3. Results

A total of 372 questionnaires were delivered and returned, with a recovery rate of 100%. Twelve questionnaires were excluded due to incomplete filling out. The rest questionnaires were valid (360), with an effective rate of 96.8%.

Table 1 presents the sociodemographic characteristics of the midwives. 350 midwives were women with only 10 being men. Nearly half of the midwives (49.4%) were aged ≤ 29 years old. Over half of the midwives had given birth and had received education majored in midwifery. The number of midwives from the general hospitals (*n* = 186, 51.7%) is a little more than that of the maternal and child hospitals (*n* = 174, 48.3%). Most midwives were from the labor rooms of tertiary hospitals (74.2%), had a primary professional title (73.1%) and a bachelor's or above degree (78.3%), and received training on maternal birthing positions (73.6%).

### 3.1. Midwives' Knowledge, Attitudes, and Practices of Liberal Birthing Position Management During the Second Stage of Labor

Table 2 presents the mean scores of the midwives' knowledge, attitudes, and practices of liberal birthing position management during the second stage of labor in mainland China.

The midwives had a high level of knowledge with a mean total score of 23.28 (SD = 4.58) out of 28, which meant an 83.1% rate of correct answers. The dimension that had the lowest rate of correct answers was Indication (72.7%), followed by the Contraindications (75.2%), the Concept (89.5%), and the Function (91.8%).

The total mean attitude score was 43.06 (SD = 9.08) out of 76, which was a lower moderate level. The total mean score of the Interest and Applying Tendency dimension 23.20(±3.86) out of 28 was higher than that of the Worry dimension 19.86 (±7.92) out of 48. The three items that had the lowest mean scores were “*I am concerned that medical disputes will arise if there is an event of a bad outcome in a non-lying delivery*” [1.19 (±0.92)]; “*I am worried that when the mother adopts a sitting, squatting, or standing position to deliver, I won't be able to care for the baby in time and the baby will fall to the floor*” [1.34 (±1.02)]; and “*I am worried that it is not convenient to disinfect and spread a towel when taking sitting, squatting, and standing birthing positions, and it is easy to pollute*” [1.37 (±0.99)].

The mean total score of the midwives' practices was 3.03 (±1.85) out of 6, indicating a moderate level.  Table 3 presents the midwives' practices of liberal birthing position management during the second stage of labor. More than one-fifth of the midwives had never practiced it before. Although 77.5% of the midwives had helped the women to adopt the liberal birthing positions, less than half (42.5%) were able to practice independently. 63.6% of the midwives had tried three, or above positions. However, most midwives (95%) used lithotomy position in the phase of the actual birth of the baby when the baby actually emerges.

### 3.2. The Relationships Among Midwives' Knowledge, Attitudes, and Practices of Liberal Birthing Position Management During the Second Stage of Labor

Table 2 also presents the relationships among midwives' knowledge, attitudes, and practices of liberal birthing position management during the second stage of labor. There is a positive correlation between the total score of knowledge and attitude (*r* = 0.37, *p* < 0.001) as well as practices (*r* = 0.41, *p* < 0.001). Additionally, there is a positive correlation between the total score of attitude and practice (*r* = 0.40, *p* < 0.001).

### 3.3. The Predictors of Midwives' Practices of Liberal Birthing Position Management During the Second Stage of Labor

A total of 12 variables were included in the multiple linear regression model, including statistically significant variables in the univariate analysis (age, fertility status, highest education, hospital category, hospital grade, professional title, position, experience of training on the maternal birthing position, and experience of clinical teaching), plus knowledge score, attitude score, and the midwifery working years. The regression analysis revealed that six variables (experience of training on the maternal birthing position, hospital grade, experience of clinical teaching, attitude score, knowledge score, and hospital category) were the predictors and jointly explained the variation of 48.4% (Table 4).

## 4. Discussion

This study examined the midwives' knowledge, attitudes, and practices of liberal birthing position management during the second stage of labor and identified the predictors of practice in mainland China. The study found that although midwives had positive attitudes to learn and practice, their practices were limited.

The midwives had good knowledge of the Concept and Function dimensions of liberal birthing position management during the second stage of labor. However, their knowledge of Indications and Contradictions required improvement. This result was consistent with a previous qualitative study, which found that Chinese midwives' perceptions of the indications and contraindications of liberal birthing positions were divergent and ambiguous [29]. Considering that 73.6% of the midwives in the present study had received training on maternal birthing positions during the second stage of labor, it may be related to the limited content that midwives have learned in the past, mainly reflected in: Firstly, currently, in Chinese undergraduate midwifery teaching materials, it is recommended to use a specific position during the first stage of labor only in cases of abnormal fetal position, and there are no specific implementation methods and precautions [30]. Secondly, although there are topics on free-position childbirth in academic conferences, it is basically the sharing of implementation effects, and there are no specific practical courses; the previous study [29] also suggested that midwives may lack the skills in practices of liberal birthing position management during the second stage of labor. Thirdly, there are no targeted systematic training and education programs based on the current state of midwives' knowledge and attitudes. As such, it is essential to review and revise the current training content and methods.

The present study also found that midwives were worried about the practice of liberal birthing position management during the second stage of labor, which was consistent with the results of the previous studies [31]. The midwives worried about the possible risks in the practical operations. The lowest scored item “*I am concerned about medical disputes will arise if there is an event of a bad outcome in a non-lying delivery*” indicated that midwives were very worried about the consequences of a bad outcome. It may be because the liberal birthing position is quite a new intervention in midwifery practice and is not incorporated into routine care in mainland China. Considering the attitudes were correlated with the knowledge in the present study, the midwives' lack of knowledge of indications and contraindications may have contributed to this. The findings of the present study suggested that it is necessary to improve the midwives' attitudes by enhancing their relevant knowledge and skills.

The present study found that although most midwives could encourage women to change positions during the second stage of labor, their practice of liberal birthing position management during the second stage was limited. Most of them used lithotomy position in the phase of actual birth of the baby when the baby actually emerges. Only 5% could use the lateral lying position, standing position, sitting position, and hands and knees position (Table 3). This result was consistent with the results of three recent domestic surveys in mainland China. Scholars investigated 90, 50, and 154 medical institutions in Henan Province, Sichuan Province, and Shandong Province and found that hospitals that carried out free birth positions only accounted for 35.8%, 44%, and 44.7%, respectively [32–34].

The results of the present study were compared with those from other countries. A survey showed that the more commonly used positions in the phase of the actual birth of the baby when the baby emerges in the UK were the lateral position, hands-and-knees position, and kneeling position. In France, 39.5% of births are in a sitting position, and 8.8% are in a kneeling position. In Canada, some regions have a high rate of liberal birthing positions up to 63.3%. The difference in the midwifery practice of liberal birthing position management during the second stage of labor may be due to the fact that China lacks an authoritative program to guide midwives to implement [35].

In this study, work experience and continuing education training in obstetrics were not included as predictors of behavioral scores, and the greatest contribution to behavioral scores was having received training on maternal birthing positions (38.0%, Table 4), suggesting the importance of specialized training. Several studies [36–39] have shown that specific training, intensive training, and job-oriented training can improve behavior levels. Furthermore, the midwives' knowledge and attitudes also predicted their practices of liberal birthing position management during the second stage of labor. These findings were consistent with the KAP theory that knowledge affects attitude and is the foundation of attitude and practice, and attitude affects practice also [40, 41]. Therefore, it is necessary to strengthen the special training of liberal birthing position management and focus training on the knowledge and attitudes that are lacking in the assessment. The present study also found that the hospital grade, hospital categories, and having experience of clinical teaching were also predictors of midwives' practices of liberal birthing position management during the second stage of labor.

The midwives in the tertiary hospital had better practices than the midwives in the secondary hospital, which was consistent with previous Chinese studies [19, 42]. The reasons may be related to the positioning and learning environment of the hospital. Usually, tertiary hospitals have teaching tasks and pressure and are tasked with training lower level hospital staff and teaching interns, contributing to a better atmosphere of learning and professional development. Thus, their attitudes can be more positive, and it will be easier for midwives there to learn to improve their practices and competence. Alternatively, it may also relate to the fact that top hospitals mostly have enough equipment/space to effectively choose the birthing position [43].

The category of hospital is also significantly related to the midwives' practices. The midwives in maternal and child hospitals demonstrated better practices compared to those in general hospitals. This was possibly linked to variations in pregnant women's medical condition upon hospital admission for delivery; dedicated facilities for women and children administer fundamental medical care in the form of specialized hospitals [44]. Due to an overall lower risk profile, that is, liberal birthing positions may be more suitable for more pregnant women. The level of midwifery practice and pregnancy outcomes correlates with midwives' experience [45]. Midwives working in maternal and child hospitals have increased opportunities to learn and collect experience, particularly about indications and contraindications. Consequently, they become less apprehensive and can confidently facilitate a wider range of birthing positions.

Having experience in clinical teaching was a predictor of practice. Engaging in clinical teaching engagement has a significant impact on the core competencies of the Chinese midwifery workforce [46, 47]. Teaching and learning reinforce each other and promote their knowledge. Additionally, midwifery teachers often receive incentives that stimulate midwives' sense of achievement, work enthusiasm, and behavior level.

We had used the same questionnaire to examine 350 midwives' knowledge, attitudes, and practices of liberal birthing position management during the second stage of labor in Guangzhou, China, in 2018. The results of the present study were compared with our previous study in 2018. Unfortunately, the midwives' knowledge [23.28 (±4.58)], attitudes [43.06 (±9.08)], and practice [3.03 (±1.85)] in the present study were also the same with that of 2018 [24.09 (±4.72); 43.77 (±10.79); 3.52 (±1.58)]. In the 2018 study, predictors affecting practice also included years of midwifery service, with seven factors collectively explaining 41.1% of the variation in practice. This may be related to the fact that midwives who joined the workforce in recent years did not receive adequate training and practice during the COVID-19 pandemic. These findings suggested that midwifery managers and educators in China need to focus on strengthening the training of midwives and improving their capacity in the management of liberal birthing positions.

This study has some strengths. This study explored the factors related to the midwives' practice of liberal birthing position management during the second stage of labor, which is a significant aspect of midwifery practice. Moreover, we attempted to select various categories and grades of hospitals. Further research could be conducted on effective improvement strategies from the midwife perspective, as well as in-depth research from the expectant mothers' perspective to find the factors affecting maternal practice, to promote liberal birthing positions.

This study had limitations. Firstly, the sample was predominantly female, which reflects the gender distribution of the midwifery profession but may limit the generalizability of the findings. Secondly, although we attempted to select various categories and grades of hospital, we did not use the stratified random sampling method. The sample was mainly composed of tertiary hospitals, young midwives, midwives with higher levels of education, and experiences of clinical teaching. Future studies may increase the number of hospitals included, expand the sample size, and improve population representation. Furthermore, multiple linear regression indicates that six factors can jointly explain a 48.4% variation in the total score of practice; structural factors like the allotment of manpower and special equipment, the establishment of process guidelines, and the mechanism of performance distribution can be potentially included for examination in future research.

## 5. Conclusion

The midwives' practices of liberal birthing position management during the second stage of labor were unsatisfactory, and related factors included knowledge, attitudes, and training status in this field, and hospital grade, hospital category, whether they participate in clinical teaching. The findings of this study may guide midwifery managers and educators in developing interventions to improve midwives' practices of liberal birthing position management during the second stage of labor.

## 6. Implications

Firstly, midwifery managers should do a good job in midwives' training planning and department management to promote the practice of liberal birthing positions. Training is an important way to improve midwives' clinical practice competence, as noted by the International Confederation of Midwives in 2021. Based on the results of this study, it is suggested that midwifery managers should establish nursing learning organizations to support the development of midwives, such as giving midwives more opportunities to participate in clinical teaching or act as “teachers” and share reports regularly, organizing special case discussions in the department, arranging midwives to go to tertiary hospitals, maternal and child hospitals, or even to study and exchange overseas, so as to improve their professional competence on liberal birthing position management during the second stage of labor.

Furthermore, midwifery educators should do a good job in teaching reform to promote the practice of liberal birthing positions. A scoping review indicated that the only modified factor of the primary determinants impacting the core competitiveness of midwives is education [46]. The International Confederation of Midwives (ICM) advocates for the development of midwifery education curriculum in its report “Midwife Core Competencies 2013 Revision.” The study revealed that respondents expressed the desire for training on the content of nontraditional birthing positions during their schooling. Thus, educators in educational institutions, in conjunction with hospital experts, should establish a midwifery professional system based on the present state of midwifery education and clinical requirements. Effective integration of WHO's suggested strategies for promoting natural childbirth is imperative, alongside developing an educational curriculum consistent with international institutions' standards. Nursing organizations, such as nursing societies and education centers, can organize training courses or academic exchanges regularly to share new knowledge and skills. They should also establish special training projects aligned with the updated midwifery content, standardized midwife training, and specialized nurse training that integrates international concepts, such as liberal birthing positions, and should explore offering professional development resources, including teaching aids and simulation training facilities. In terms of content, in addition to sharing the advantages and concepts of free position delivery, it can increase the knowledge of indications and contraindications; in the form of teaching, besides teaching in classroom, simulation training, scene drills, and clinical practice can be added to improve the training effect.

## Figures and Tables

**Table 1:** Midwives' sociodemographic characteristics and the comparison of practices (*n* = 360).

Variable	*f* (%)	Midwives' practice		
		*M* ± SD	*t/F*	*p*
Age (years old)			*F* = 8.64	< 0.001
≤ 29	178 (49.4)	2.67 ± 1.92		
30–39	119 (33.1)	3.18 ± 1.72		
≥ 40	63 (17.5)	3.73 ± 1.68		
Gender			*t* = −1.78	0.076
Male	10 (2.8)	2.00 ± 1.89		
Female	350 (97.2)	3.05 ± 1.85		
Having given birth before			*t* = −2.56	0.011
Yes	195 (54.2)	3.26 ± 1.71		
No	165 (45.8)	2.75 ± 1.98		
Highest education			*t* = −4.31	< 0.001
Diploma or below	78 (21.7)	2.24 ± 1.89		
Bachelor or above	282 (78.3)	3.24 ± 1.79		
Major of graduation			*t* = 0.03	0.98
Nursing	140 (38.9)	3.03 ± 1.82		
Midwifery	220 (61.1)	3.02 ± 1.88		
Hospital category			*t* = −2.56	0.011
General	186 (51.7)	2.78 ± 1.82		
Maternal and child	174 (48.3)	3.28 ± 1.86		
Hospital grade			*t* = −5.34	< 0.001
Secondary	93 (25.8)	2.17 ± 1.90		
Tertiary	267 (74.2)	3.32 ± 1.75		
Professional title			*F* = 8.49	< 0.001
Primary	263 (73.1)	2.81 ± 1.90		
Intermediate	86 (23.9)	3.49 ± 1.63		
Senior	11 (3.1)	4.55 ± 0.69		
Position			*F* = 8.33	< 0.001
Junior nurse	211 (58.6)	2.67 ± 1.89		
Senior nurse	76 (21.1)	3.46 ± 1.64		
Group leader	53 (14.7)	3.32 ± 1.88		
Head nurse	20 (5.6)	4.35 ± 0.88		
Experience of training on maternal birthing position			*t* = −10.79	< 0.001
Yes	265 (73.6)	3.62 ± 1.48		
No	95 (26.4)	1.38 ± 1.82		
Experience of clinical teaching			*t* = −7.50	< 0.001
Yes	225 (62.5)	3.57 ± 1.63		
No	135 (37.5)	2.12 ± 1.86		

**Table 2:** The mean scores of midwives' knowledge, attitudes, and practices and their correlation analysis (*n* = 360).

Variables	*M* ± SD (Range)	Knowledge	Attitude	Practice
		*r*		
Knowledge	23.28 ± 4.58 (3–28)			
Attitudes	43.06 ± 9.08 (24–76)	0.37⁣^∗^		
Practices	3.03 ± 1.85 (0–6)	0.41⁣^∗^	0.40⁣^∗^	

⁣^∗^*p* < 0.001.

**Table 3:** Distribution of midwives' practices of liberal birthing position management during the second stage of labor (*n* = 360).

Items	*f* (%)
1. Have you helped the women to use liberal birthing position during the second stage of labor?	
No	81 (22.5%)
Yes, with other midwives' help	126 (35.0%)
Yes, by myself	153 (42.5%)
2. How many positions (lithotomy, lateral lying, sitting, squatting, hands and knees, and standing) have you tried to guide the woman during the second stage of labor?	
1	81 (22.5%)
2	50 (13.9%)
3	101 (28.1%)
4 or above	128 (35.5%)
3. What position do you often help the woman to use in the phase of the actual birth of the baby when the baby actually emerges?	
Lithotomy position	342 (95.0%)
Lateral lying position	13 (3.61%)
Sitting position	1 (0.28%)
Squatting position	0
Hands and knees position	1 (0.28%)
Standing position	3 (0.83%)

**Table 4:** The predictors of midwives' practices of liberal birthing position management during the second stage of labor (*n* = 360).

Variables	*B*	SE	*β*	95% CI	VIF	*t*	*p*
Constant	−4.13	0.61	—	−5.56∼−2.98	—	−6.76	< 0.001
Age	0.14	0.20	0.06	−0.25∼0.53	4.45	0.70	0.484
Fertility status	−0.38	0.19	−0.10	−0.77∼−0.01	1.79	−2.03	0.053
Education	0.22	0.20	0.05	−0.16∼0.61	1.30	1.15	0.253
Professional background	−0.03	0.16	−0.01	−0.34∼0.29	1.22	−0.15	0.878
Working experience	0.21	0.14	0.12	−0.07∼0.49	4.73	1.46	0.147
Hospital category	0.47	0.16	0.12	0.16∼0.79	1.30	2.90	0.004
Hospital grade	0.92	0.19	0.22	0.54∼1.29	1.40	4.89	< 0.001
Professional title	0.01	0.20	0.00	−0.39∼0.40	2.19	0.03	0.973
Position	0.06	0.12	0.03	−0.17∼0.29	2.38	0.51	0.613
Experience of clinical teaching	0.58	0.18	0.15	0.23∼0.94	1.54	3.24	0.001
Continuing obstetrics training	−0.07	0.12	−0.02	−0.29∼0.16	1.28	−0.56	0.573
Received training on maternal birthing position	1.61	0.17	0.38	1.27∼1.95	1.18	9.28	< 0.001
Knowledge score	0.06	0.02	0.14	0.02∼0.09	1.36	3.19	0.002
Attitude score	0.03	0.01	0.15	0.01∼0.05	1.31	3.38	0.001

*Note:R* ^2^ = 0.500; adjusted *R*^2^ = 0.484; *F* = 29.06; *p* < 0.001.

## Data Availability

The data that support the findings of this study are available from the corresponding author upon reasonable request.

## Supporting Information

Supporting file includes three parts: (1) Questionnaire on midwives' knowledge of liberal birthing position management during the second stage of labor; (2) Questionnaire on midwives' attitude of liberal birthing position management during the second stage of labor; and (3) Questionnaire on midwives' practice of liberal birthing position management during the second stage of labor.
