# Supplementary material for: Midwives' Knowledge, Attitudes, and Practices of Liberal Birthing Position Management During the Second Stage of Labor: A Cross-Sectional Study
Source: J Nurs Manag. 2025 Feb 24;2025:6139992. doi: 10.1155/jonm/6139992 (PMC11985226; doi:10.1155/jonm/6139992)
Supplement: Supporting Information — Additional supporting information can be found online in the Supporting Information section. [file 6139992.f1.docx]

Supplementary

Questionnaire on midwives' **knowledge** of liberal birthing position management during the second stage of labor

| **Item** | **Correct** | **Incorrect** | **Uncertain** |
| --- | --- | --- | --- |
| 1. A liberal birthing position means that the parturient chooses the position that she feels comfortable with and can effectively promote natural childbirth according to her own will, instead of lying in bed or fixing a single position |  |  |  |
| 2. During the second stage of labor, a liberal birthing position is recommended, including a half-lying position, side-lying position, lateral prone position, sitting position, squatting position, kneeling position, and standing position |  |  |  |
| 3. Half-lying position is lying on the bed on the back, but the angle between the upper body lifting and the bed surface is between 45-90 degrees |  |  |  |
| 4. The lateral prone position is lying on the bedside, but the back is not vertical to the bed surface, the lower leg is straight, the upper leg flexion is more than 90 degrees, and the body is like a rotating axis, turning forward incompletely |  |  |  |
| 5. The propped forward sitting position is a position where the woman sits with her legs flat on her back. the body leaning forward, the arms on the thighs or the support frame, or riding on the chair, stool, and toilet, and supporting the upper body on the back of this equipment |  |  |  |
| 6. Squatting position refers to woman's feet flat on the floor or bed, lowering the body and looking for a companion or railing or other methods to maintain body balance |  |  |  |
| 7. The propped forward kneeling position refers to woman's knees on the bed or the floor, and her body can lean forward on the back of the bed, chair, delivery ball, or other supports, and the knees can be protected with soft pillows and cushions |  |  |  |
| 8. Hand and knee position refers to woman's knees on the bed or the floor, leaning forward, landing hands or fists on the ground to support the body. Soft pillows and cushions can be used to protect both knees and hands |  |  |  |
| 9. Knee chest position refers to woman's knees and forearms on the ground and the chest should be close to the bed surface or floor as far as possible. Soft pillows and cushions can be used to protect the knees and elbows |  |  |  |
| 10. Standing position refers to woman standing beside the bed, holding the bed bar with both hands, slightly opening the legs, and slightly shaking the buttocks from left to right. It can also make the maternal body lie forward on the support, such as the companion, the higher bed, or the delivery ball. |  |  |  |
| 11. It can guide the adjustment of body position according to the degree of self-tolerance and comfort. When the labor progress is slow, guide the puerpera to change the position about 5-6 contractions, and the time for each position should not exceed 30 minutes |  |  |  |
| 12. Sitting on the delivery ball or standing position with the body leaning forward can guide the puerpera to cooperate with the pelvic swing |  |  |  |
| 13. The liberal birthing position can improve maternal-fetal circulation and reduce the incidence of fetal distress and neonatal asphyxia |  |  |  |
| 14. The liberal birthing position can facilitate the rotation of the fetal head, facilitate maternal exertion, promote the progress of the labor process, and reduce the rate of cesarean section |  |  |  |
| 15. The liberal birthing position can improve maternal self-control consciousness, relieve pain, and improve comfort (such as side-lying position, half-sitting position, kneeling position, or standing position) |  |  |  |
| 16. The liberal birthing position can reduce the rate of episiotomy. |  |  |  |
| 17. When suspected or diagnosed as persistent occiput posterior position, side-lying position, lateral prone position, standing position, anteversion position, hand knee position, and knee-chest position can be adopted |  |  |  |
| 18. If the fetal head is large, the head and pelvis are not evenly inclined, the posterior occipital position or transverse occipital position, and the pelvic joint needs more variability, it can adopt the forward tilt squat position, the supporting type forward tilt sitting position, the standing position and walking position, and the pelvis swing |  |  |  |
| 1. When the fetal head position is higher, and fetal distress occurs in the lateral or supine position, a propped forward kneeling position can be adopted |  |  |  |
| 20. For patients with prolapse or compression of the umbilical cord, cervical edema or slow regression of anterior lip, anteversion kneeling position, hand knee position, or knee-chest position can be adopted |  |  |  |
| 21. When sedative and epidural analgesics are used, it is safe to take the half-lying position, side-lying position, and half-sitting position |  |  |  |
| 22. When the labor progress is too fast, a side-lying position can be used for easy management |  |  |  |
| 23. When the head of the fetus is crowning and contractions are occurring, women delivering in vertical positions, such as standing or sitting, should not hold their breath and push |  |  |  |
| 24. Squatting, kneeling, and standing positions are not suitable for women who feel fatigued, have numbness in their legs, or have knee pathologies |  |  |  |
| 25. The vertical position is not suitable for women with hypertension |  |  |  |
| 26. When the fetal position is known or suspected to be an occiput posterior position, it is not suitable to adopt the semi-sitting position |  |  |  |
| 27. Occipitoposterior position and hypotension should not be used in a semi-recumbent position |  |  |  |
| 28. If the fetal membrane has been broken and the fetal head is not connected, it is not suitable for pregnant women |  |  |  |

Questionnaire on midwives' **attitude** of liberal birthing position management during the second stage of labor

| **Item** | **Strongly disagree** | **Disagree** | **Uncertain** | **Agree** | **Strongly agree** |
| --- | --- | --- | --- | --- | --- |
| 1. I agree that in addition to the needs of the disease, we should not fix the single body position during delivery and try to adopt a comfortable position (i.e., liberal birthing position) |  |  |  |  |  |
| 2. I think the effect of the liberal birthing position management during the second stage of labor is reliable |  |  |  |  |  |
| 3. I think the advantages of the liberal birthing position management during the second stage of labor outweigh the disadvantages |  |  |  |  |  |
| 4. I am interested in a liberal birthing position management during the second stage of labor |  |  |  |  |  |
| 5. I will discuss the liberal birthing position with others |  |  |  |  |  |
| 6. I will recommend and guide the mother to deliver in a free position. |  |  |  |  |  |
| 7. I would like to know more about the liberal birthing position |  |  |  |  |  |
| 8. I am worried that the lack of leg separation in the side-lying position would affect the descent of the fetus |  |  |  |  |  |
| 9. I am worried that pushing in a non-supine position won't be very effective |  |  |  |  |  |
| 10. I am concerned that in the non-recumbent position, the mother will be too tired or have difficulty maintaining her balance so she may fall easily |  |  |  |  |  |
| 11. I am concerned about the risk of umbilical cord prolapse when the pregnant with broken water is in a liberal birthing position |  |  |  |  |  |
| 12. I am worried that the perineum will tear easily when sitting, squatting, and standing |  |  |  |  |  |
| 13. I am worried that it is not convenient to monitor the fetal heart rate in a non-supine position |  |  |  |  |  |
| 14. I am worried that it is not convenient to observe the visible fetal head in a non-supine position |  |  |  |  |  |
| 15. I am worried that it is not convenient to disinfect and spread a towel when taking sitting, squatting, and standing birthing positions, and it is easy to pollute |  |  |  |  |  |
| 16. I am worried that when the mother takes a sitting, squatting, or standing position to deliver, I won’t be able to care for the baby in time and the baby will fall to the floor |  |  |  |  |  |
| 17. I am worried that I'll feel pain in the back and waist when woman laboring in a non-supine position |  |  |  |  |  |
| 18. I am worried that women would not agree to the implementation of a liberal birthing position |  |  |  |  |  |
| 19. I am concerned that medical disputes will arise if there is an event of a bad outcome in non-supine delivery |  |  |  |  |  |

Questionnaire on midwives' **practice** of liberal birthing position management during the second stage of labor

1. Have you helped the women to use liberal birthing positions during the second stage of labor?

① No.

② Yes, with other midwives’ help

③ Yes, by myself

(2) How many positions have you guided the woman during the second stage of labor? **(multiple choices, you can refer to the following diagram）**


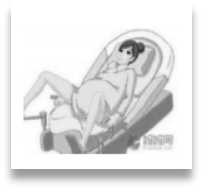

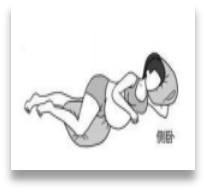

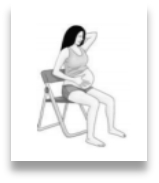


lithotomy position lateral lying positions sitting position


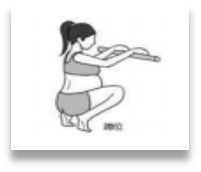

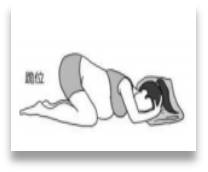

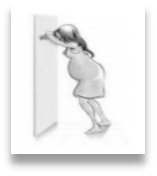


squatting position hands and knees position standing position

① 1

② 2

③ 3

④ 4 or above

(3) Which position do you often help the woman to use in the phase of the actual birth of the baby when the baby actually emerges?

① Lithotomy position

② Lateral lying positions

③ Sitting position

④ Squatting position

⑤ Hands and Knees position

⑥ Standing position
